# Innovative Use of Coronary GuideLiner to Facilitate Patent Ductus Arteriosus Stent Reintervention

**DOI:** 10.1016/j.jscai.2025.103576

**Published:** 2025-04-15

**Authors:** Bayan Issa, Marjan Hesari, Brent M. Gordon, Justin R. Ryan, Anna Hedberg, Clinton Fulk, Ryan Reeves, Howaida El-Said

**Affiliations:** a Department of Pediatrics, University of California San Diego, La Jolla, California; b Division of Cardiology, Rady Children’s Hospital, San Diego, California; c Helen and Will Webster Foundation 3D Innovations Lab, Rady Children’s Hospital, San Diego, California; d Department of Neurological Surgery, UC San Diego Health, La Jolla, California; e Sulpizio Cardiovascular Center, University of California San Diego, La Jolla, California

**Keywords:** coronary GuideLiner, patent ductus arteriosus stent reintervention, patent ductus arteriosus stenting

## Abstract

This is the first report using a coronary GuideLiner (Teleflex) to overcome the challenge of patent ductus arteriosus (PDA) stent crossing and restenting. We present 2 patients, a 3-month-old and a 15-month-old, with complex cyanotic heart disease and ductal-dependent pulmonary blood flow who previously underwent PDA stenting and required reintervention on the PDA stent. Historically, patients who require reintervention for PDA stenting have been challenging, especially when restenting is needed. The use of the GuideLiner was a game-changer in these 2 cases.

## Introduction

Infants with complex congenital heart defects with ductal-dependent blood flow often require multiple interventions. Patent ductus arteriosus stenting (PDAS) is evolving as the procedure of choice to establish pulmonary blood flow until definitive repair or second stage palliation can be achieved.^1^ PDAS has a higher incidence of reintervention than Blalock-Taussig shunt.^2^ However, PDAS allows further dilation of the stent for growth if more time is needed before definitive repair or palliation. Reintervention for PDAS is particularly challenging when restenting is required. Here, we illustrate the novel use of a coronary GuideLiner (Teleflex) to overcome these obstacles.

### Case 1

**Figure 1:**
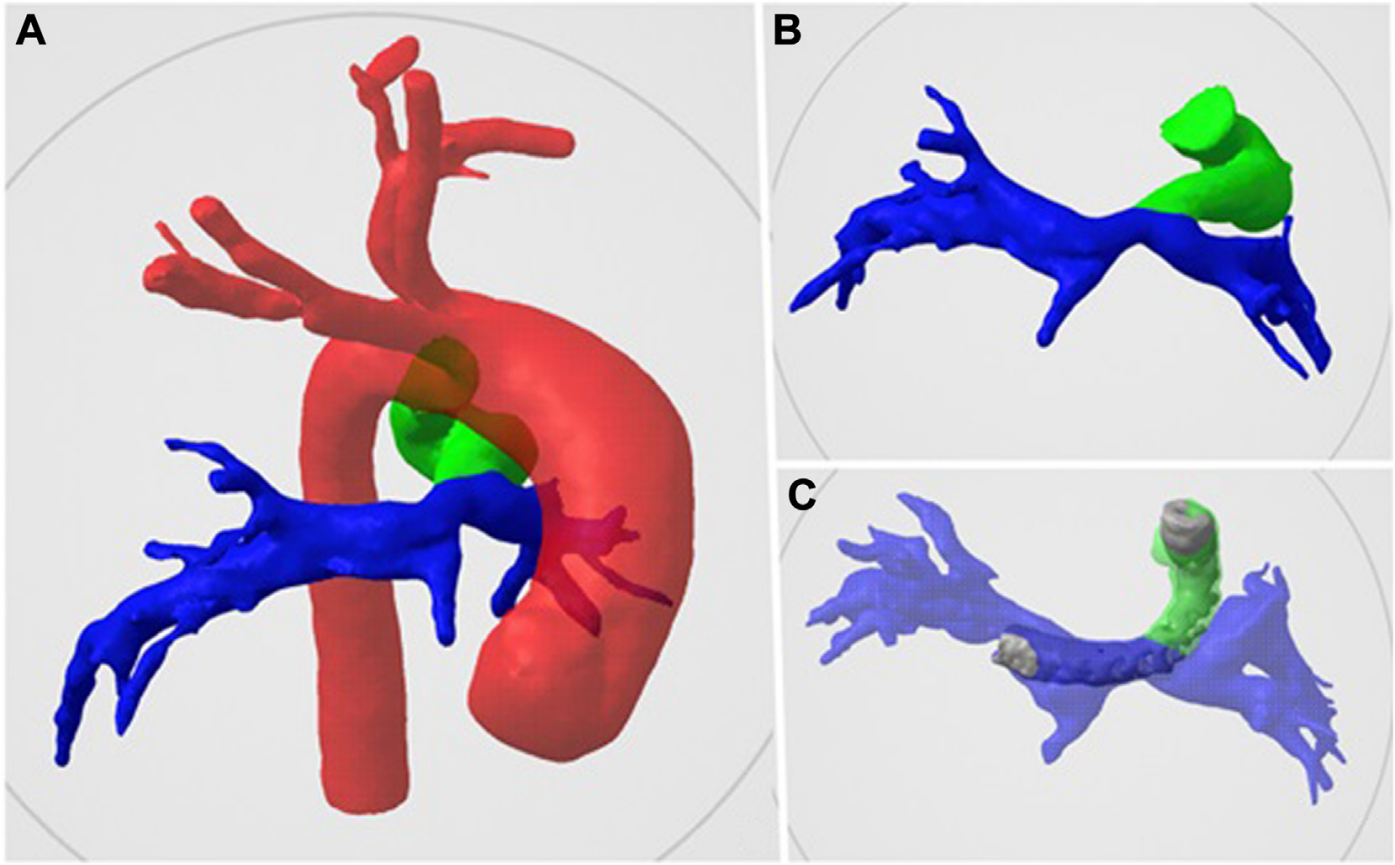
**3DReconstruction of case 1.(A, B)** A type 3 tortuosity patent ductus arteriosus (PDA) inserting into the left pulmonary artery (LPA), with the proximal LPA likely incorporating PDA tissue. (**C**) Post-PDA stenting, showing the stent extending into the right pulmonary artery with an acute angle jailing the LPA, while maintaining good distal LPA flow.

A 3-month-old male infant with dextrocardia, double inlet left ventricle, pulmonary atresia, and a tortuous type III patent ductus arteriosus (PDA) underwent PDAS with a 3.5 mm × 38 mm Onyx Frontier stent (Medtronic) at 6 days of life. He later developed progressive hypoxemia and was taken to the catheterization laboratory for evaluation. Angiography revealed in-stent stenosis; however, further intervention was deemed challenging due to the stent’s acute angle (Figure 1). To improve blood flow, a GuideLiner was used to help cross the existing stent and place a 4.5 mm × 15 mm Onyx Frontier stent.

#### Procedure details

##### GuideLiner catheter design

**Figure 2:**
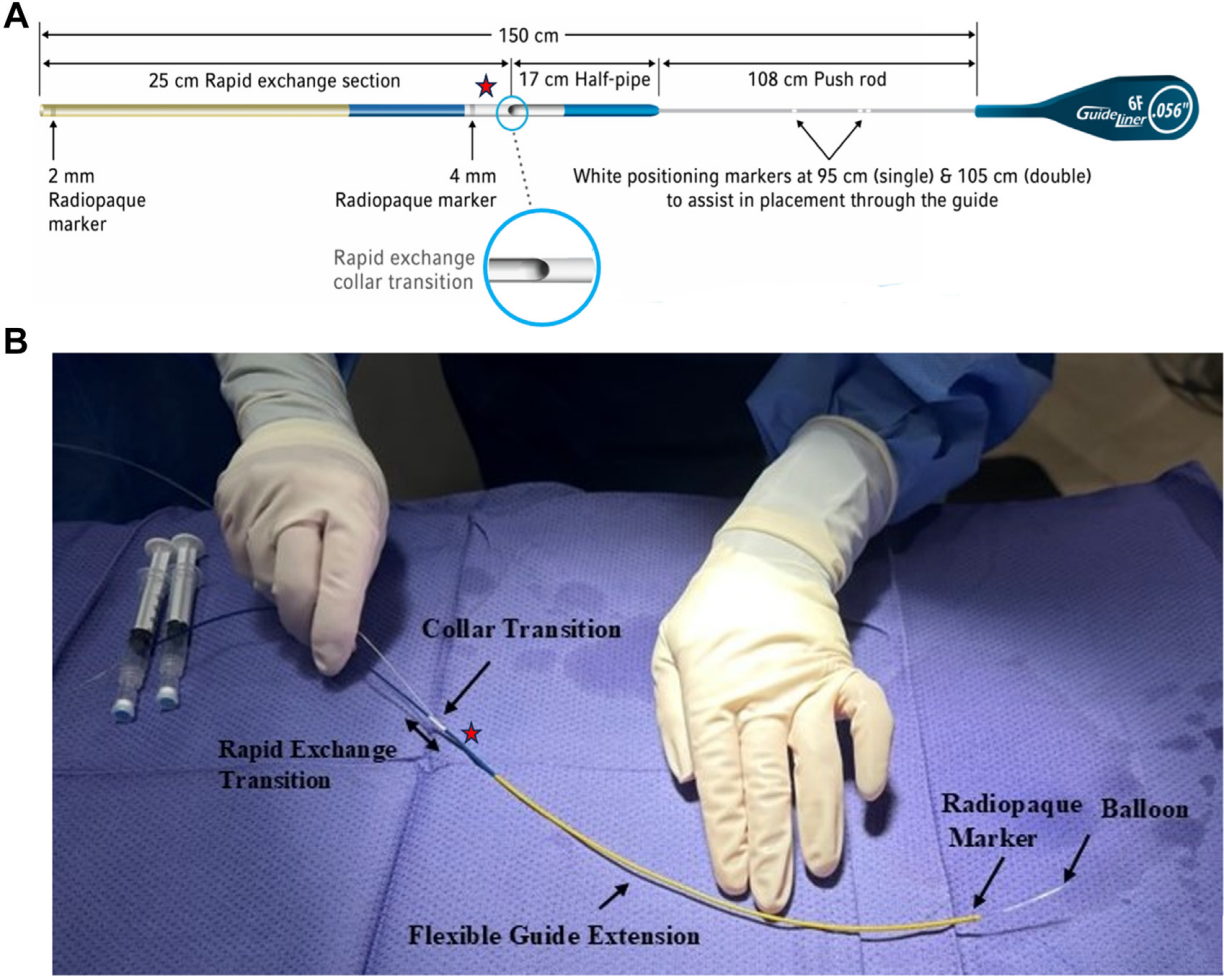
**GuideLiner catheter.(A)** Schematic image courtesy of Teleflex.^3^ (**B**) Assembly of the GuideLiner catheter system components. The red star indicates the clamp placement to prevent bleeding.

The GuideLiner catheter has a long metal shaft connected to a 20-cm flexible catheter, with either silicon or hydrophilic coatings (Figure 2).^3^ The device comes in 5F, 5.5F, 6F, 7F, and 8F sizes. The outer diameter (OD) of the 5.5F GuideLiner is 4.8F (0.063 inches), so it does not fit through a 4F sheath but fits easily through a 5F sheath (this was tested). However, the OD of the 5F GuideLiner is 4F (0.053 inches), and it can theoretically fit through a 4F sheath and accommodate a 4.5-mm stent (OD 3.2F or 0.042 inches). However, this configuration was not used in our case and was not bench-tested. The GuideLiner is typically inserted over a guide wire and advanced to the distal edge of the guide catheter. A balloon catheter is then advanced on the guide wire, passing through the proximal port of the guide extension. The balloon is then advanced into the vessel, allowing the atraumatic advancement of the GuideLiner.

##### Preparation

1. Bench testing

We bench-tested the 5.5 GuideLiner and determined that it could fit in a 5F but not a 4F Prelude Ideal sheath (Merit Medical). Although not stated in the instructions for use, our testing confirmed that clamping the GuideLiner with a coronary wire inside did not compromise lumen integrity or damage the wire after clamp removal. Clamping is essential when using a short sheath, as it enables balloon and stent exchange while minimizing bleeding from the GuideLiner’s collar. Additionally, we verified that the wire, balloon, and GuideLiner remained stable and did not twist during the procedure (Figure 2, Video 1).

2. We used 3-dimensional (3D) segmentation of the computed tomography scan to assess the trajectory and determine the ideal stent length (Figure 1).

##### Procedure

1. Sheath and wire placement
  • A 5F × 11 cm Prelude Ideal sheath was placed in the left carotid artery. A Steri-strip was placed on the short sheath to mark its position.
  • A 4F angled glide catheter (Terumo), a Renegade microcatheter (Boston Scientific), and a 300 cm HI-TORQUE Balance Middle Weight Universal II Guide wire (Abbott) were inserted coaxially through the 5F sheath.
  • After multiple attempts to enter the central portion of the stent, we decided to cross the stent via a side strut and the HI-TORQUE Balance Middle Weight Universal II Guide wire was advanced into the distal left pulmonary artery (LPA) across the side cell.
2. Dilation and GuideLiner placement

We placed a 2-mm balloon inside the GuideLiner and advanced them together over the wire through the 5F sheath. We inflated the 2-mm balloon to dilate the proximal and distal cells but could not pass the GuideLiner over it. The 2-mm balloon was removed over the wire. We then clamped the GuideLiner with a hemostat artery forceps over the wire to prevent bleeding from the proximal opening (collar transition) of the GuideLiner during balloon exchange. This maneuver enables the operator to work through a short sheath, eliminating the need for a long sheath (>25 cm) to control bleeding at the transitional collar site (Figure 2).

**Figure 3:**
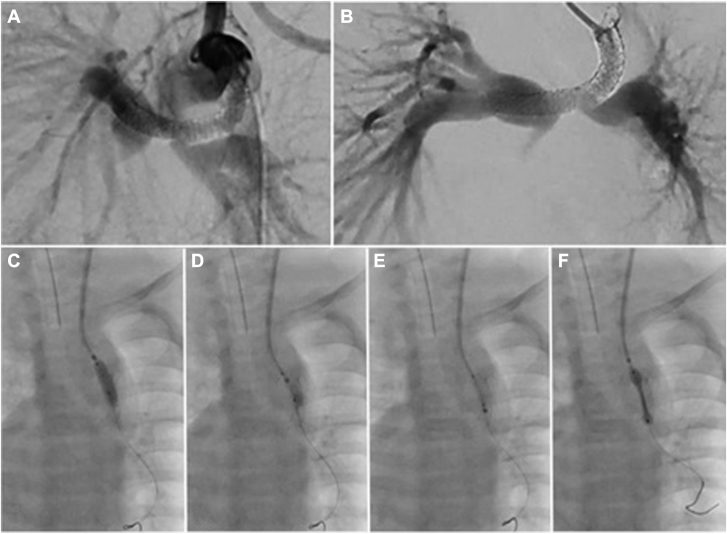
**Angiographic images from case 1.(A)** The acute angle of the patent ductus arteriosus stent with stent fracture and intimal buildup, **(B)** improved caliber of the stent and flow in the left pulmonary artery (LPA) postintervention, **(C)** HI-TORQUE Balance Middle Weight Universal II Guide wire placed across the strut of the stent into the LPA–the GuideLiner is parked above the stent and the 4.5-mm balloon is inflated in the stent’s proximal strut to dilate it, **(D)** the 4.5-mm balloon inflated with half of it in the GuideLiner and the other half in the stent, **(E)** Advancement of the GuideLiner over the balloon as it is being deflated, (F) GuideLiner pulled back and stent being deployed.

Next, we placed a 4.5 mm × 12-mm balloon inside the GuideLiner over the wire. With the GuideLiner parked at the origin of the stent in the aorta, the 4.5-mm balloon was passed through the stent strut into the LPA and was used to dilate the proximal strut, the portion of the stent in the PDA, the strut leading to LPA, and the LPA itself. We then pulled the 4.5-mm balloon halfway into the PDA stent and halfway into the GuideLiner, allowing for inflation with half of the balloon inside and half outside the GuideLiner. As the 4.5-mm balloon gradually deflated, the GuideLiner was successfully advanced over the balloon and into the stent (Figure 3 and Video 2).

3. Stent deployment

We deployed a 4.5 mm × 15-mm stent through the GuideLiner. During exchanges, the GuideLiner was clamped over the wire.

4. Wire repositioning and further dilation

The GuideLiner was removed, and the wire was repositioned to address the narrowing at the LPA/right pulmonary artery (RPA) junction. A 4.5-mm balloon was used to dilate the entire stent complex. An exit angiogram confirmed stent patency before sheath removal.

## Case 2

**Figure 4:**
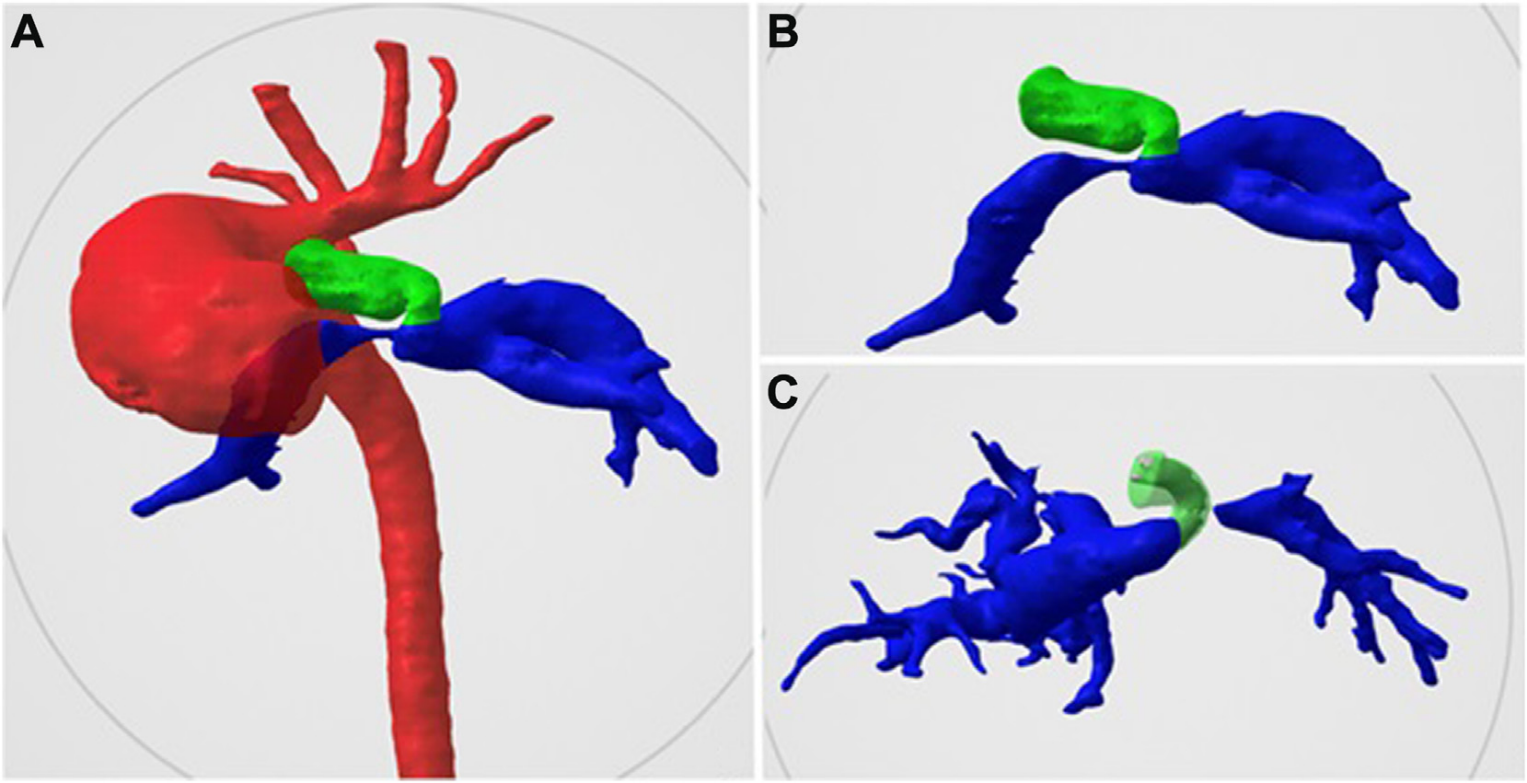
**Three-dimensional reconstruction illustrating case 2.(A, B)** A type 2 tortuosity patent ductus arteriosus with proximal right pulmonary artery stenosis before patent ductus arteriosus stenting (PDAS); **(C)** post-PDAS, showing a stent fracture with stenosis at the origin of the jailed proximal left pulmonary artery, with a well-sized distal vessel.

**Figure 5:**
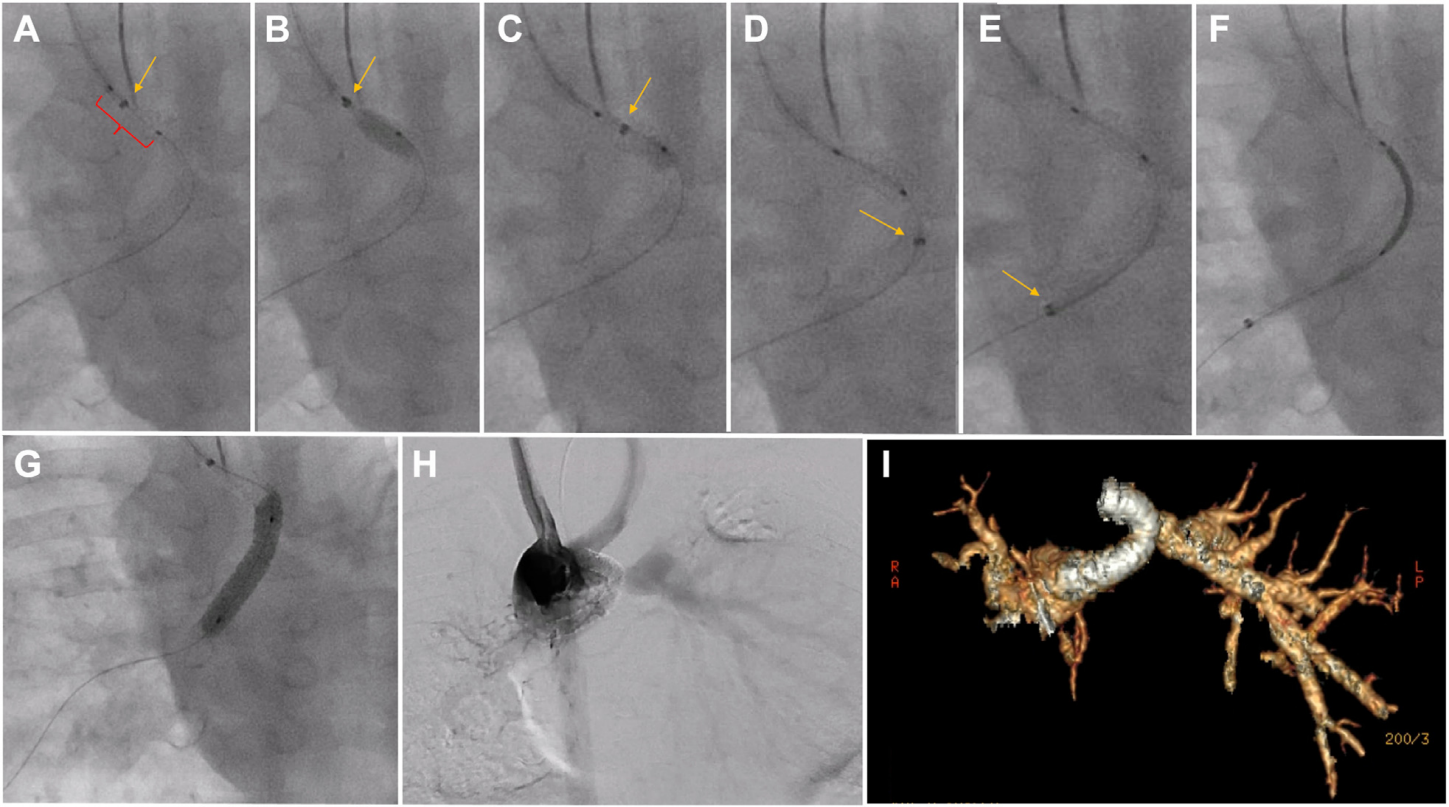
**A step-by-step illustration of GuideLiner use during restenting of the patent ductus arteriosus stent in case 2.** (**A-C**) Demonstrate the balloon-assisted technique to advance the GuideLiner. (**D, E**) depict the GuideLiner successfully advancing across the previously placed stent. (**F**) shows the stent positioned within the GuideLiner, and (**G**) illustrates the stent being inflated within the existing stent. (**H**) Subtraction angiogram performed after stent placement shows flow into the left pulmonary artery (LPA) through the stent struts. (**I**) highlights a 3-dimensional segmentation from a computed tomography scan conducted 6 months later, demonstrating adequate LPA growth. The yellow arrow indicates the marker on the tip of the GuideLiner, and the red bracket outlines the balloon before inflation within the GuideLiner.

A male infant with a double outlet right ventricle, unbalanced atrioventricular canal, malposed great arteries, pulmonary atresia, and RPA narrowing underwent PDAS with a 3 mm × 30 mm Onyx Frontier stent at 90 days of age (Figure 4). At 8 months, he presented with hypoxia and was found to have in-stent stenosis. Using the GuideLiner technique described above, a 4.5 mm × 18 mm Onyx Frontier stent was placed in the PDA, extending into the stenotic RPA, intentionally jailing the LPA, leading to clinical improvement. The final angiogram confirmed a widely patent PDA stent and branch pulmonary arteries (Figure 5 and Video 3).

## Discussion

Managing infants with complex congenital heart defects requires innovative approaches to address and facilitate difficult procedures, PDAS plays a crucial role in optimizing outcomes.^4^^,^^5^ However, these procedures can be complicated by stent fracture or intimal buildup, which may necessitate further interventions. Anatomical and procedural complexities, including tortuous arterial ducts, increase the risk of reintervention on PDAS.^6^

The GuideLiner catheter, commonly used for complex coronary interventions, provides deeper target vessel intubation than may be achieved with a guide catheter.^7^ Common indications include increasing support and stability, facilitating balloon and stent crossing through complex anatomy. It is highly effective in coronary tortuosity, severe calcifications, and aneurysms in adults, with success rates of up to 93%, demonstrating its safety and efficacy despite occasional complications.^8^, ^9^, ^10^, ^11^ The softness and flexibility of the GuideLiner make it uniquely suited for PDA stenting in which the use of a long sheath to cross the lesion can disrupt the PDA and is not recommended. Our cases demonstrated that the GuideLiner provides significant benefits for challenging PDA restenting, preventing distortion and facilitating smoother advancement.

## Conclusion

This is the first report of using a coronary GuideLiner in PDA restenting in infants with complex ductal-dependent congenital defects. With some minor modifications, we found the GuideLiner to be a valuable and important piece of equipment in the interventionalist’s toolbox.

## Supplementary material

Video 1 Illustrating bench testing of the GuideLiner and the balloon.

Video 2 Illustrating catheterization procedure with angiograms during GuideLiner use for patent ductus arteriosus stent crossing and re-stenting.

Video 3 Illustrating the balloon-assisted technique used to advance the GuideLiner® through the previously placed stent.
